# D2B-Functionalized Gold Nanoparticles: Promising Vehicles for Targeted Drug Delivery to Prostate Cancer

**DOI:** 10.1021/acsabm.2c00975

**Published:** 2023-02-09

**Authors:** Monira Sarkis, Georges Minassian, Nadim Mitri, Kamil Rahme, Giulio Fracasso, Roland El Hage, Esther Ghanem

**Affiliations:** † Department of Sciences, Notre Dame University-Louaize, 72 Zouk Mosbeh, Lebanon; ‡ School of Chemistry & AMBER Centre, University College Cork, T12 YN60 Cork, Ireland; § Department of Medicine, University of Verona, I-37134 Verona, Italy; ∥ Laboratory of Physical Chemistry of Materials (LCPM), PR2N (EDST), Faculty of Sciences II, Lebanese University, Campus Fanar P.O. Box 90656, 1103 Beirut, Lebanon; ⊥ Polymers Composites and Hybrids (PCH), IMT Mines Ales, 30100 Ales, France; # biobank.cy-Center of Excellence in Biobanking and Biomedical Research, Molecular Medicine Research Center, University of Cyprus, 1678 Nicosia, Cyprus

**Keywords:** gold nanoparticles, D2B, bioconjugation, targeted delivery, prostate cancer

## Abstract

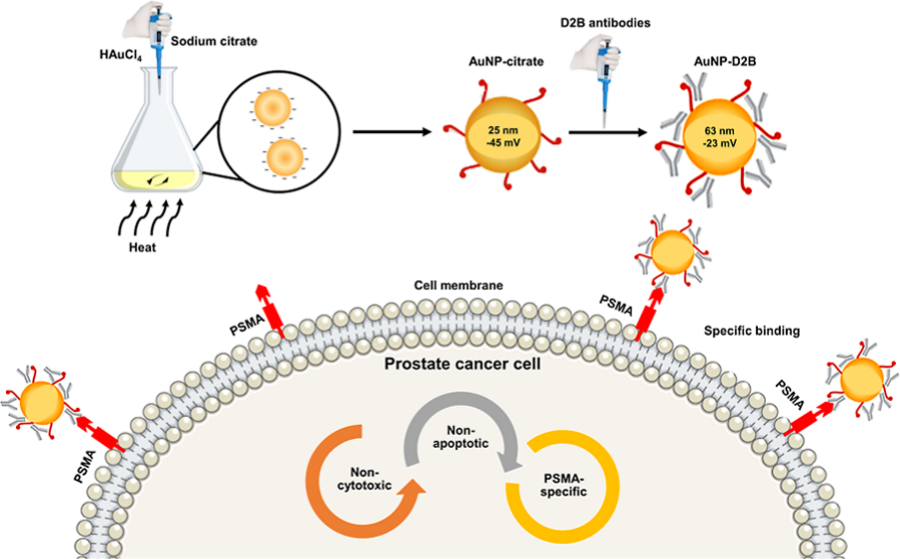

Despite the multitude of therapeutic agents available to treat prostate cancer (PC), there are still no effective and safe measures to treat the tumor. It remains a challenge to develop a simple approach to target PC with specific antibodies. In our study, D2B monoclonal antibodies against a prostate-specific membrane antigen (PSMA) were used. We investigated the functionalization of gold nanoparticles (AuNPs) with D2B to generate favorable physicochemical and biological properties that mediate specific binding to PC. For this purpose, AuNPs with a size of about 25 nm were synthesized in water using sodium citrate as a reducing and stabilizing agent and then coated with D2B. Major physicochemical properties of naked and D2B-coated AuNPs were investigated by ultraviolet–visible (UV–vis) spectroscopy, dynamic light scattering (DLS), and zeta potential measurements. The successful binding of D2B to AuNPs-citrate caused a 15 nm red shift in the UV–vis. This was assessed by DLS as an increase in zeta potential from ∼−45 to ∼−23 mV and in the size of AuNPs from ∼25 to ∼63 nm. Scanning electron microscopy confirmed the size shift of AuNPs, which was detected as an exterior organic layer of D2Bs surrounding each AuNP. Even at high exposure levels of the bioconjugates, PSMA-PC-3 cells exhibited minimal cytotoxicity. The specific and dose-dependent binding of AuNPs-D2B to PC-3-PSMA cells was validated by flow cytometry analysis. Our data provide effective drug delivery systems in PC theranostics.

## 1 Introduction

Prostate cancer (PC) is still one of the most lethal tumors in males.^1^ Current cancer treatments have a low curative effectiveness in patients with metastatic PC.^2^ Chemotherapy administered systemically^3^ has unfavorable adverse effects on healthy tissues.^4,5^ As a result, scientists are continuously on the search for specific PC cell markers to create effective tailored treatment.

Prostate-specific membrane antigen (PSMA), a cell surface marker overexpressed in nearly all PC stages, is increased by more than 10-fold in aggressive PC.^6−8^ PSMA is as an enzyme associated with PC cell proliferation.^9^ It activates the oncogenic signals responsible for stimulating the metabotropic glutamate receptors via vitamin B9 cleavage, making it a promising target for photothermal and photodynamic therapy.^10^ The majority of PSMA is internalized into the endosomal compartment following antibody binding to its extracellular portion.^11,12^ Antibodies differ in many ways, most notably in their mode of action, stability, solubility, affinity, and specificity.^13,14^ Monoclonal antibodies (mAbs) against PSMA, such as murine J591, 7E11, and human MDX-070,^9,15^ have shown promising outcomes in PC theranostics. Another form of the anti-PSMA mAb, namely, D2B, which is generated by conventional hybridoma technology,^16^ outperforms PSMA targeting capabilities^17^ with efficient D2B–PSMA complex internalization.^18−20^ On the other hand, D2B has drawbacks such as delayed blood clearance, unspecific background activity, and limited tumor penetrability and accumulation. For example, D2B in the ^111^In-DTPAD2B-IRDye800CW shows undesirable liver accumulation regardless of its successful uptake by PC tumors.^21^ Similarly, ^111^In-DTPA-D2B-IRDye700DX presents high spleen uptake with additional non-hepatotoxic uptake by the liver.^22^

In our previous study, we reviewed the versatile applications of the D2B mAb in PC.^23^ The compiled findings confirm that D2B complexation with nanocarriers improves its performance and diminishes its setbacks.^23^ A spectrum of D2B nanocarriers exists, such as gold nanoparticles (AuNPs-TR-SH-D2B-PEG),^24^ carbon nanohorns (f11-CNHs),^25^ pegylated radioimmunoconjugates (^223^RaA-silane-PEG-D2B),^26^ and chitosan NPs (CS-D2B).^27^ AuNPs demonstrated significant translational applications in cancer therapy^28−32^ including PC^33^ due to their small size,^34^ biocompatibility,^35^ and optimum biodistribution in vivo.^35^ Furthermore, the strong affinity of AuNPs for the amino, phosphate, and thiol groups of numerous molecular probes facilitates the accommodation of large payloads.^36,37^ However, previous studies either combined AuNPs with other NP types or relied on laser ablation, thiolation, PEGylation, or more complex synthesis methods to generate D2B-NPs, including AuNPs.^23^ Nonetheless, AuNPs remain attractive candidates for the prospective synthesis of D2B-coated AuNPs, assuming that no additional polymers or sophisticated synthesis procedures are used.^23^

In this study, we are the first to investigate the potential of producing D2B-conjugated gold-citrate NPs (AuNPs-D2B) using a simple and cost-effective method while testing their binding specificity to PSMA-expressing PC-3 cells (PC3-PSMA). In our design model, AuNPs capped with citrate (AuNPs-citrate) were synthesized in water using sodium citrate as a reducing and stabilizing agent and then coated with D2B antibodies. Several methods, including UV–vis spectra, scanning electron microscopy (SEM), dynamic light scattering (DLS), and zeta potential measurements, were used to characterize AuNPs before and after coating with the D2B mAb. In addition, water-soluble tetrazolium salt 1 (WST-1) cell proliferation and DNA fragmentation assays were used to investigate the cytotoxicity of AuNPs-D2B on PC-3 and PC-3-PSMA cell lines. Finally, flow cytometry and fluorescence-activated cell sorting (FACS) were used to determine the successful PC-3-PSMA cellular binding capacities and specificities of these bioconjugates. Our collected data present a simple, yet effective coating method for AuNPs with D2B or another antibody, offering promising nanovehicles for PC-targeted therapy.

## 2 Materials and Methods

### 2.1 Chemicals

Purified H_2_O with an approximate electrical resistivity of 18.2 MΩ cm was used as a solvent. The glassware was cleaned with aqua regia (3 parts of concentrated HCl and 1 part of concentrated HNO_3_), rinsed with distilled water, ethanol, and acetone, and oven-dried before use. Tetrachloroauric acid trihydrate (HAuCl_4_, 3H_2_O), sodium citrate dihydrate (C_6_H_5_Na_3_O_7_·2H_2_O), and sodium hydroxide were purchased from Sigma-Aldrich. The WST-1 cell proliferation assay kit was purchased from Roche. Radioimmunoprecipitation assay buffer (RIPA) lysis buffer (50 mM NaCl, 25 mM Tris HCL (pH 8), 0.5% sodium deoxycholate, and 0.5% Triton).

### 2.2 Synthesis of Gold Citrate Nanoparticles

To a 100 mL round-bottom flask containing 48 mL of deionized water at 95 °C, 100 mL of NaOH (0.1 M) and 0.961 mL of HAuCl_4_ (13 mM) were added under stirring, followed by the fast injection of 0.625 mL of sodium citrate (0.1 M). Upon the injection of sodium citrate, the color of the solution changed from pale yellow to colorless within 2 min, then to gray after about 5 min, and then shifted to clear red that deepened with time to deep red-wine (∼30 min). The solution was kept under gentle stirring for another 30 min after the color stabilized (deep red-wine).

### 2.3 Synthesis of D2B-Coated Gold Citrate Nanoparticles

To a 50 mL beaker containing 10 mL of the AuNPs-citrate pristine solution in an ice bath, 400 μL of D2B solution (0.099 mg/mL) was added dropwise with constant stirring. The color of the solution changed gradually from deep red-wine to pink-violet within an hour, indicating that D2B is successfully grafted onto the AuNPs’ surface. The amount of D2B attached to AuNPs was estimated via a pre-calculation based on our previous work published by Rahme et al.^38^

### 2.4 UV–Vis Spectroscopy Analysis

Optical spectra were obtained using a UV/Vis Analytik Jena SPECORD 250 PLUS spectrophotometer (300–900 nm range, 0.5 nm resolution).

### 2.5 DLS and Zeta Potential Measurements

The size distribution and surface charge (zeta potential) of AuNP colloidal solutions were determined by DLS using the Malvern Zetasizer Nano-ZS (model ZEN3600; Malvern Instruments Inc., Westborough, MA, USA) using the default NIBS 173° back-scattering technique. The model used in the fitting procedure was based on Mark Houwink’s parameters. The data were adjusted using the cumulative fit given by the suppliers. Measurements were performed on the pristine solutions of AuNPs (∼50 μg/mL) using disposable folded capillary cuvettes at 25 °C. For easier comparison, each sample was repeated three times.

### 2.6 Scanning Electron Microscopy

AuNPs coated with D2B colloidal solution were deposited on a silicon wafer and dried in air prior to inspection by SEM.^39^ The sample was inspected using a Hitachi S-4300 environmental scanning electron microscope operating at 10 kV. The sample was metalized with carbon to avoid charging during observation.

### 2.7 Cell Lines and Culture Conditions

Wild-type PC-3-PSMA negative and PC-3-PSMA transfected positive cell lines were cultured in RPMI 1640 (Sigma-Aldrich) supplemented with 10% fetal bovine serum and antibiotics (100 U/mL penicillin and 100 μg/mL streptomycin) in a humidified atmosphere containing 5% CO_2_ at 37 °C. Cells were grown as a monolayer (70–80% confluence) before being incubated with various concentrations of NPs.

### 2.8 Antibodies

The D2B monoclonal antibody (anti-PSMA)^40^ was utilized to generate AuNPs-D2B; fluorescein-isothiocyanate (FITC)-labeled monoclonal goat anti-mouse IgG was utilized as the secondary antibody (ab6785, Abcam, USA); Hoechst 33258 nucleic acid stain (ab228550, Abcam, USA) was also used.

### 2.9 WST-1 Cell Proliferation Assay

PC-3-PSMA cells at the log phase were seeded in a 96-well plate at a seeding density of 5 × 10^4^ cells/mL. After overnight incubation, the cells were treated for 4 h with increasing concentrations of naked AuNPs-citrate (control group) or AuNPs-D2B (C1 = 20 μg/mL, C2 = 12 μg/mL, C3 = 6 μg/mL, C4 = 3 μg/mL, and C5 = 1 μg/mL). Stock solutions of 50 μg/mL AuNPs-citrate and 250 μg/mL AuNPs-D2B were used to reach the tested concentrations in a final volume of 200 μL/well. Dilutions were mixed in 90% RPMI media and 10% sterile deionized water using the following formula *C*_stock_ × *V*_stock_ = *C*_well_*V*_well_. All wells were treated with 10 μL of the WST-1 reagent for 2 h. Results were read at 450 nm using the MultiGo-Scan ELISA reader.

### 2.10 DNA Fragmentation

Phenol/chloroform (1:1) was used to extract DNA from AuNP-treated cells (a stock solution of 250 μg/mL AuNPs-D2B was used for the dilutions using the following formula: *C*_stock_ × *V*_stock_ = *C*_well_ × *V*_well_). Cells were vortexed in RIPA buffer for efficient lysis and protein solubilization and then centrifuged at 8000*g* for 1 min at RT. The resulting top aqueous phase was transferred to a fresh tube. Then, 500 μL of the DNA-containing solution was pelleted with 3 M sodium acetate and 2 mL ethanol and centrifuged for 15 min at top speed. The resulting pellet was resuspended in 60 μL of a deionized water-RNase solution (0.4 mL water +5 μL of RNase), and the quantity and quality of DNA were assessed by Nanodrop and analyzed by agarose gel (1%) electrophoresis (∼20 ng/mL of DNA per lane).

### 2.11 Flow Cytometry

The cells were harvested with 0.025% Trypsin/0.01% w/v EDTA/PBS for 3 min. The cells were then washed and treated with the NPs at 37 °C or at 4 °C for 4 h. A stock solution of 250 μg/mL AuNPs-D2B was used to form different concentrations using the following formula: *C*_stock_ × *V*_stock_ = *C*_well_ × *V*_well_. After washing with 1× PBS, bound AuNPs-D2B were stained with goat anti-mouse IgG Ab conjugated to FITC in the dark for 1 h. Then, the cells were washed and stained with Hoechst33258 to exclude cell debris. Samples were read using a PARTEC Cube 8 flow cytometer (Scientific & Technical Supplies Co.). At least 50,000 cells were gated and further analyzed using the FlowJo software (FlowJo, LLC, Ashland, OR). Data were normalized using a basic statistical formula: normalized value , where min(*x*) corresponds to the lowest value of the control data set, max(*x*) corresponds to the highest value of the highest AuNPs-D2B concentration data set, and (*x*_i_) stands for the value to be normalized.

### 2.12 Statistical Analysis

Data were reported as mean ± SEM (standard error of the mean), analyzed by one-way ANOVA, and differences between the tested and the control groups were assessed by post hoc and Tukey’s tests. The statistical significance was set at *p* < 0.05, and each experiment was performed and validated at least three times. Significance was indicated in each graph, with (*) for a *p*-value <0.05, (**) for a *p*-value <0.01, and (***) for a *p*-value <0.001. ns corresponds to non-significant difference.

## 3 Results

### 3.1 Synthesis and Characterization of AuNPs-Citrate and AuNPs-D2B

**Figure 1:**
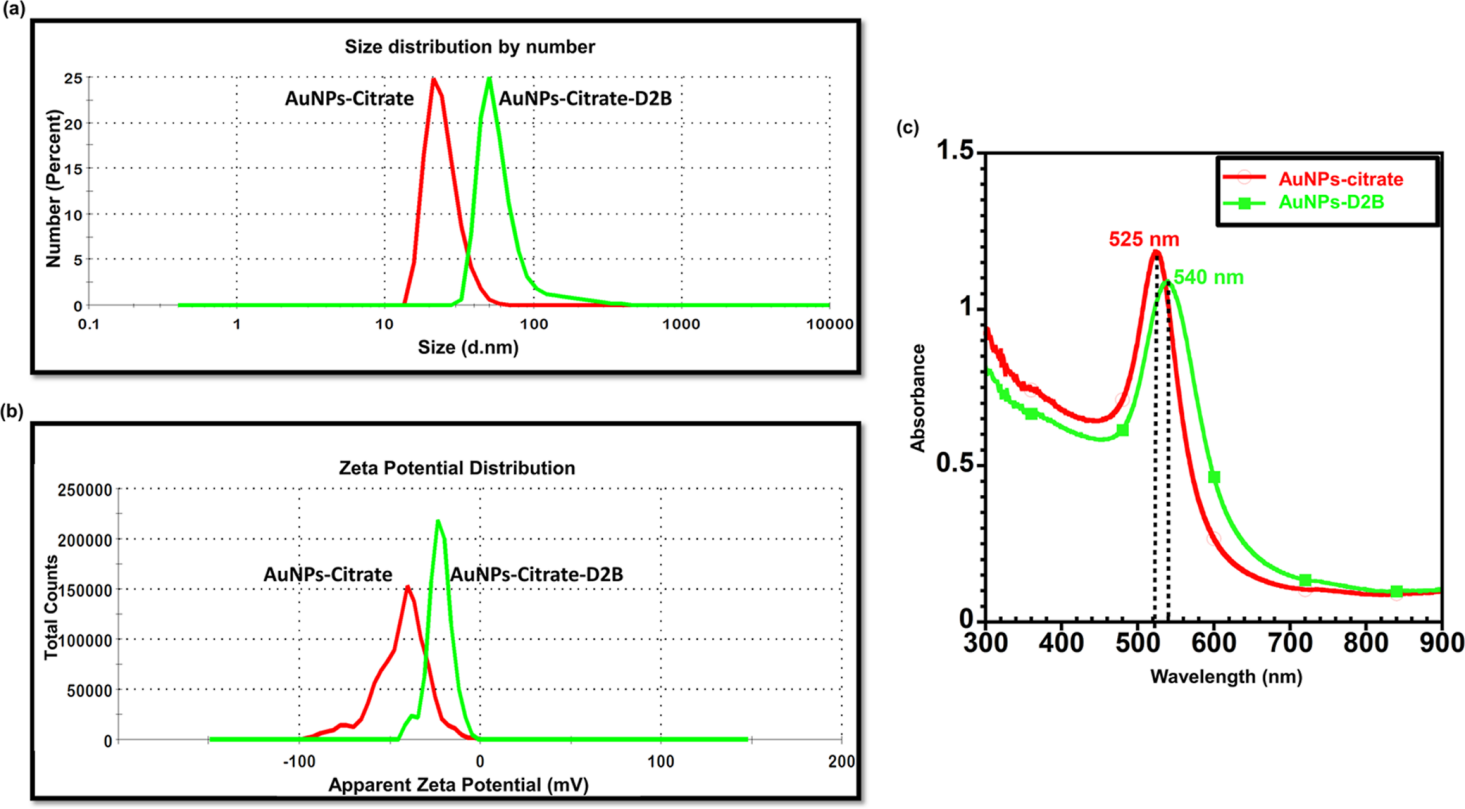
DLS size distribution by number, zeta potential, and UV–vis spectra measurements of AuNPs-citrate and AuNPs-D2B. (a) DLS size distribution by number and (b) zeta potential measurement of AuNPs-citrate before and after bioconjugation with D2B. A zeta potential charge shift from ∼−45 mV for AuNPs-citrate to ∼−23 mV for AuNPs-D2B and an increase in the AuNPs size from 25 ± 0.6 nm to 62 ± 1.5 nm confirmed the successful conjugation of AuNPs-citrate with D2B. The size variation is not quantitative and does not reflect the molar ratio of D2B to AuNPs. (c) UV–vis spectra of AuNPs-citrate (∼25 nm) before and after bioconjugation with D2B. The addition of D2B to the AuNPs-citrate colloidal solution caused a red shift (higher wavelength) of about 15 nm in the UV–vis spectra. Abbreviations. AuNPs: gold nanoparticles; DLS: dynamic light scattering; UV–vis spectra: ultraviolet visible spectra.

**Figure 2:**
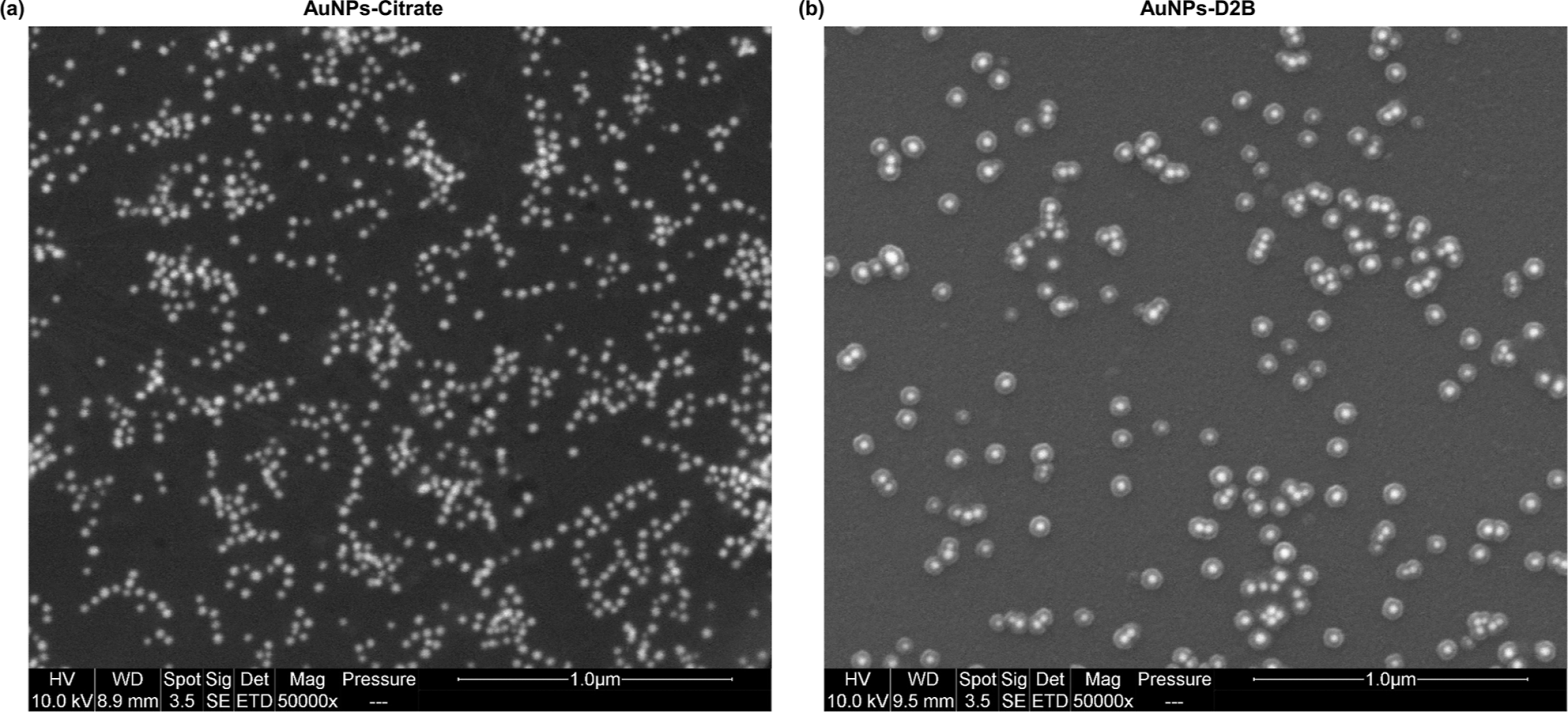
SEM images of AuNPs-citrate and AuNPs-D2B. (a) As analyzed by Image J software (scale bar 1 μm), the SEM image of AuNPs-citrate pristine solution shows that the AuNPs are well dispersed and spherical in shape with a diameter of 24 ± 5 nm. (b) A representative SEM image of AuNPs-D2B revealed that the 24 ± 5 nm AuNPs cores are coated with a D2B corona layer of approximately 25 ± 2 nm thickness (scale bar, 500 nm). Abbreviations. AuNPs: gold nanoparticles; SEM: scanning electron microscopy.

The UV–vis spectra measurement of the obtained AuNPs-citrate colloidal solution peaked at 525 nm (Figure 1c). The synthesized AuNPs-citrate were monodispersed and spherical in shape, as shown in the representative SEM image (Figure 2a). Furthermore, Image J software analysis of the AuNPs-citrate SEM image (scale bar 1 μm) revealed an AuNPs-citrate particle size of 24 ± 5 nm (Figure 2a). Similarly, DLS size distribution by number measurements further confirmed the 25 ± 0.6 nm size of AuNPs-citrate (Figure 1a) and reported a particle charge of ∼−45 mV (Figure 1b).

On the other hand, the successful coating of AuNPs-citrate with D2B mAbs was confirmed by a red shift (higher wavelength) of about 15 nm (peaking at 540 nm) in the UV–vis spectra (Figure 1c). Also, an increase in the size of AuNPs from 25 ± 0.6 to 62 ± 1.5 nm was obtained by DLS size distribution by number measurements (Figure 1a). Similarly, zeta potential measurements revealed an AuNPs charge shift from ∼−45 mV for AuNPs-citrate to ∼−23 mV for AuNPs-D2B (Figure 1b). Finally, SEM images of AuNPs-D2B confirmed the clear coating of AuNPs with a D2B corona layer with a thickness of about 25 ± 2 nm (Figure 1b).

### 3.2 WST-1 Cell Proliferation Assay

**Figure 3:**
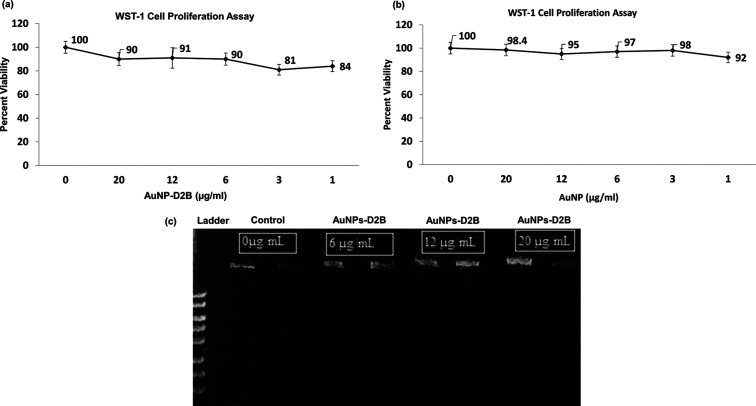
PC-3-PSMA cell proliferation and DNA fragmentation analysis at various concentrations of AuNPs-D2B vs naked AuNPs. (a,b) The tested concentrations varied between 1 and 20 μg/mL. Cells were seeded onto a 96-well plate and cultured for 24 h. Prior to exposing cells to the WST-1 reagent, cells were treated with various concentrations of AuNPs-D2B for 4 h and the absorbance was read at 450 nm. AuNPs-citrate and AuNPs-D2B showed non-cytotoxic effects on PC-3-PSMA cells, with cellular viability above 80% for all the tested concentrations, reaching (a) 90% for AuNPs-D2B and (b) 98.4% for naked AuNPs at highest concentrations. Mean ± SEM (standard error of the mean) of collected values is presented (*n* = 3). (c) Duplicates of each concentration (0, 6, 12, and 20 μg/mL) were used to determine whether AuNPs-D2B induced apoptosis in PC-3-PSMA cells. The results show that increasing AuNPs-D2B concentrations did not fragment DNA, and all samples showed patterns like the control. Abbreviations. AuNPs: gold nanoparticles; PC: prostate cancer; PSMA: prostate-specific membrane antigen; WST-1: water-soluble tetrazolium salt; SEM: standard error of the mean.

To investigate whether our synthesized AuNPs-D2B and naked AuNPs affect the proliferation profile of PC cells, the tetrazolium salt WST-1 assay was used. WST-1 is cleaved to a soluble formazan by a cellular mechanism that occurs primarily at the cell surface.^41^ This reduction is largely dependent on the glycolytic production of NAD(P)H in viable cells. The various concentrations of naked and D2B-coated AuNPs were selected with respect to those previously used in the literature.^42,43^ Moreover, naked AuNPs were considered as controls. Both naked AuNPs and AuNPs-D2B exhibited non-cytotoxic effects on PC-PSMA cells, as evidenced by a cellular viability above 80% for all the tested concentrations. Even at the highest concentration of AuNPs, cell viability was comparable to the control, with 90 and 98.4% viability for AuNPs-D2B and naked AuNPs, respectively (Figure 3a,b).

### 3.3 DNA Fragmentation

To further confirm the non-cytotoxic and non-apoptotic effects of AuNPs-D2B, genomic DNA was extracted from PC-3-PSMA cells treated with various concentrations of AuNPs-D2B (0 and 20 μg/mL). Various concentrations of AuNPs-D2B were selected with respect to concentrations previously used in the literature.^42,43^ The genome remained intact in all tested groups of different AuNPs-D2B concentrations, as confirmed by the DNA bands above the 1 kb DNA ladder and by the absence of DNA cleaved segments (Figure 3c). Thus, the physicochemical properties of AuNPs-D2B and the exposure treatment do not induce genotoxicity even at high doses.

### 3.4 Flow Cytometry Analysis

**Figure 4:**
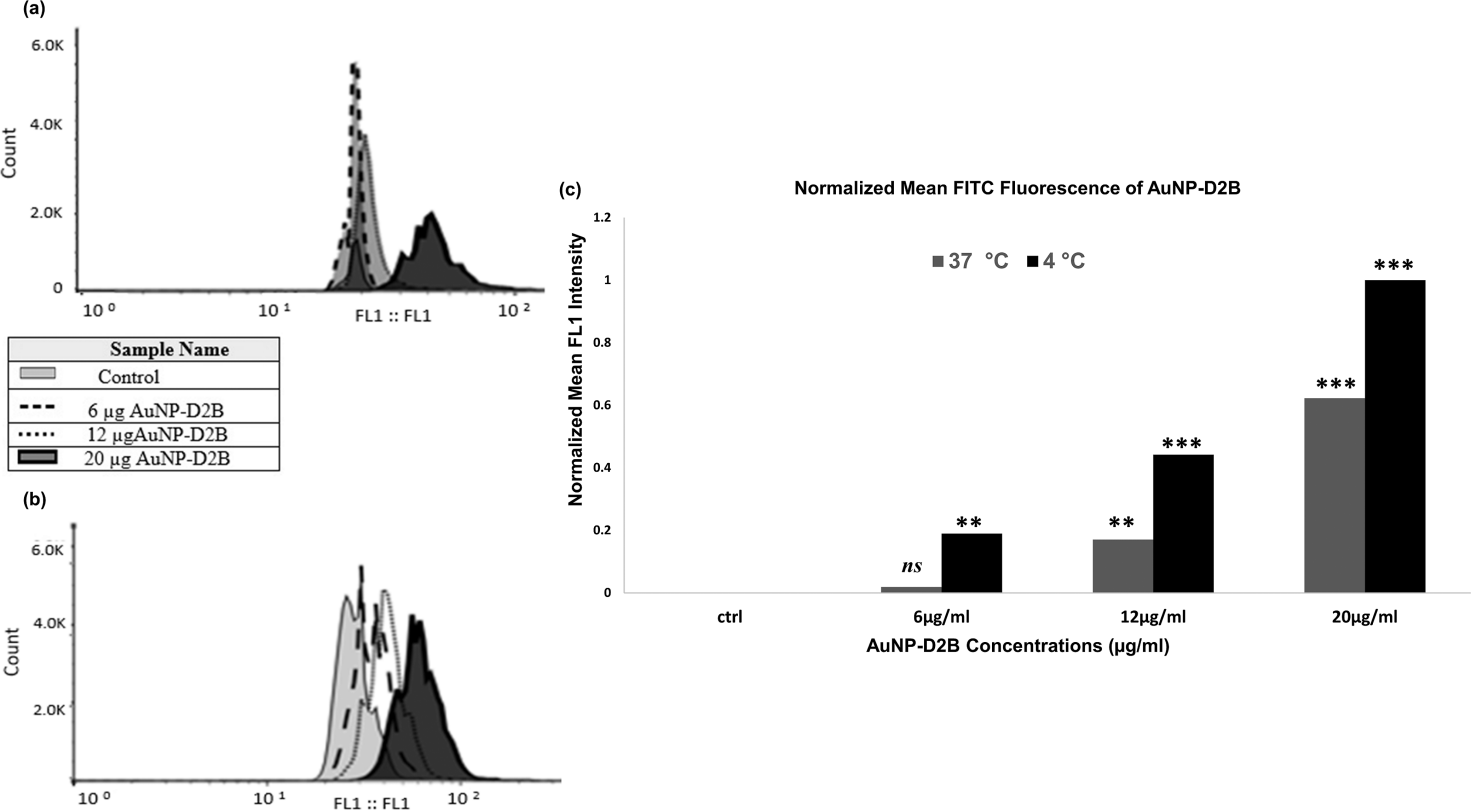
Flow cytometry analysis and FACS of AuNPs-D2B binding to PC-3-PSMA cells. PC-3-PSMA cells (2 × 10^5^) were incubated with increasing concentrations of AuNPs-D2B (0, 6, 12, and 20 μg/mL) for 4 h. Samples were then read by flow cytometry using goat anti-mouse IgG secondary antibodies conjugated to FITC (1:500). The control consisted of untreated PC-3-PSMA cells in RPMI media. A typical histogram representing the fluorescence distribution of gated PC-3-PSMA cells using logarithmic scale at (a) 37 °C and (b) 4 °C, respectively. (c) A bar graph depicting the normalized FL1 intensities of the PC-3-PSMA cells treated with AuNPs-D2B at 37 and 4 °C. Plots were gated at 50,000 cells per sample. The data show a typical experiment (*n* = 3), with (*) indicating a *p*-value of 0.05, (**) a *p*-value of 0.01, and (***) a *p*-value of 0.001. ns indicates non-significant difference. Abbreviations. AuNPs: gold nanoparticles; FACS: fluorescence-activated cell sorting; FL1: fluorescence channel 1; PC: prostate cancer; PSMA: prostate-specific membrane antigen.

Next, we investigated the binding of AuNPs-D2B to the overexpressed PSMA ligand on the PC-3-PSMA cells. The results showed a clear change in FITC fluorescence especially when cells were treated with the highest dose of AuNPs-D2B (20 μg/mL). Remarkably, low concentrations ranging between 6 and 12 μg/mL showed a similar background signal as that of untreated cells (Figure 4a,c). When 12 and 20 μg/mL of AuNPs-D2B were added, a significant increase in the mean fluorescence intensity [fluorescence channel 1 (FL1)] by 20 and 30% was observed, respectively. Exposing cells to a low dose of 6 μg/mL of bioconjugates showed a non-significant intensity.

To control the fate of the surface-bound complexes of AuNPs-D2B/PSMA, we repeated the same experiment at 4 °C. Interestingly, the results show a more intense and prominent increase in the FL1 signal even at low concentrations of AuNPs-D2B. The FL1 signal increased significantly to 39, 51, and 78% with AuNP concentrations of 6, 12, and 20 μg/mL, respectively (Figure 4b,c). For better visualization of the data, FL1 values were plotted with a clear observation of the fold increase difference between doses at different temperatures (Figure 4c).

The results confirm successful binding and imply that the higher the AuNPs-D2B concentration, the greater the number of bound PSMA receptors in PC cells. The difference in band intensity with temperature shift most likely delineates the internalization of PSMA-bound forms.

## 4 Discussion

This study reports the successful synthesis of ∼25 nm AuNPs-citrate conjugated with D2B mAbs for specific delivery to PC cells. The obtained UV–vis spectra measurements clearly show a single plasmon absorbance band for AuNPs-citrate (Figure 1c). This confirms a highly stable mixture of spherical Au NPs without aggregates.^44,45^ Also, synthesizing small-sized AuNPs ∼25 nm (Figures 1a and 2a) was performed in light of studies that have reported a correlation between NP toxicity and size^33,46^ while taking into account the need to maintain a small AuNP size after coating with the D2B antibody. Moreover, some studies have also reported high toxicity of positively charged AuNPs upon coating with minimal levels (0.05 μM) of the surfactant, cetyltrimethyl ammonium bromide (CTAB). Coating of AuNPs-CTAB with BSA, on the other hand, effectively reduced their toxicity.^47−50^ Therefore, our negatively charged AuNPs-citrate, at pH ∼7 and zeta potential of ∼−45 mV, can easily avoid unwanted charge and coating-mediated cytotoxicity. This is consistent with the results of Vijayakumar et al., 2012, where AuNPs-citrate did not induce MCF-7 and PC-3 cell cytotoxicity.^51^ In fact, citrate acts as a reducing agent by forming a layer of negative citrate ions on the AuNP surface,^52^ thus inducing sufficient electrostatic repulsion between individual particles to keep them well dispersed in the medium and biocompatible. As a result, this method produces uniform and fairly spherical NPs^51^ suitable for coating with D2B mAbs.

Citrate on the surface of AuNPs promotes antibody binding via ligand exchange, stabilizes the AuNPs, and extends their shelf life.^53^ Interestingly, non-specific adsorption of D2B onto AuNPs takes place while maintaining the negative charge and stability of the particles in colloidal solution.^54^ Furthermore, D2B can attach to the surface of AuNPs via non-covalent and covalent adsorption modes. The first mode involves ionic interactions between the negative citrate on the AuNP surface and the positively charged amino acids or the N-terminal of D2B. The second mode is through the hydrophobic interaction between D2B and the Au (metal) surface of AuNPs.^54^ As for the covalent interaction, a physical interaction occurs between AuNPs and the free sulfhydryl groups of the D2B mAb.^54^

The increase in the UV–vis spectra maximum absorption and the color shift of the AuNPs pristine solution (Figure 1c) might be attributed to the change in the refractive index around the AuNPs,^55,56^ thus confirming the attachment of D2B onto the AuNP surface.^57^ Also, the successful coating is confirmed by the ∼37 nm size increase of AuNPs (from DLS) (Figure 1a) and the formation of a ∼25 nm D2B-based organic layer surrounding the AuNP surface (from SEM) (Figure 2b). Moreover, the zeta potential charge shift of the AuNPs of ∼22 mV upon D2B addition validates the successful coating (Figure 1b). This is not surprising as the incorporation of an additive such as proteins will result in a decrease in the overall surface negativity of the AuNPs-citrate and therefore a successful conjugation.^58^ Similar size and charge variations upon antibody conjugation onto AuNPs were reported in the literature.^59,60^ Also, the final size of AuNPs-D2B of ∼62 nm remains favorable given that NPs with a size of ∼55 nm have demonstrated the fastest time of wrapping and engulfment by the cell membrane, with particles engulfed separately. For smaller NPs, they are most likely clustered before uptake, resulting in delayed uptake.^61^

The amount of D2B attached to AuNPs was estimated based on our previous work published by Rahme et al., whereby different molecular weights of thiolated polyethylene glycol (PEG) polymers were quantified on the AuNP surface using thermal gravimetric analysis and transmission electron microscopy (TEM).^38^ Hence, in the current study, the number of ∼25 nm AuNPs was estimated at ∼3.19 × 10^11^ AuNPs/mL, while D2B was used at ∼1.061 × 10^13^ molecules/mL. Therefore, the applied D2B/AuNPs ratio was 1.36:1.

The obtained proliferation assay and DNA fragmentation results (Figure 3) revealed that AuNPs-D2B are non-cytotoxic and non-apoptotic in PC-3 cells. We hypothesize that the slightly lower PC-3 cell viability in the AuNPs-D2B-treated group is a positive indicator owing to increased AuNP penetration into the cell due to D2B on its surface. However, the higher PC-3 cells’ viability of naked AuNPs results from its lower penetration ability into PC-3 cells. In contrast to previous analysis of DNA fragmentation, naked AuNPs-citrate induce cytotoxicity in human lung carcinoma type II epithelial cells (A549) and breast cancer cells (MCF-7).^42,43^ Our findings further support the notion that our nanovehicles do not induce undesired PC-3 cell cytotoxicity and apoptosis prior to cargo delivery.

The physicochemical binding properties and specificity of AuNPs-D2B for PSMA-expressing PC were also assessed. Similar to previously reported data, in vitro results showed targeted delivery of bound D2B in PSMA-PC-3 cells but not in wild-type PC cells.^4^ Interestingly, investigations of the binding and internalization efficiency of D2B-coated nanocarriers in PC cells were previously performed using flow cytometry analysis, mainly in CNHs^25^ and CS-Ab nanostructures.^27^ As a result, we used the same method to assess the extent of AuNPs-D2B-targeted internalization by PC-3-PSMA cells. Moreover, we compared the uptake of AuNPs-D2B by PC-3-PSMA cells at both 4 and 37 °C (Figure 4). It is worth noting that an incubation period of 4 h is critical since extending the timing up to 48 h induces apoptosis in A549 cells but not in MCF-7 cells.^42^ Using a different cell type, such as ovarian cancer cells (SKOV3), would allow us to better understand whether the cytotoxicity and apoptotic events caused by our synthesized AuNPs are cell-dependent.

Saturation of bound forms of PSMA-D2B-AuNPs was detected by an increase in the fluorescence signal of D2B on the surface of PC-3-PSMA tumors in a dose-dependent manner. Thus, D2B binding affinity is maintained and is not affected by AuNP conjugation, while no signal was detected with uncapped AuNPs. Shifting cells to 4 °C for 4 h or using colchicine (microtubule inhibitor) would impede or halt receptor-mediated endocytosis, consequently providing a more intense protein signal.^62,63^ Indeed, protein signals at 4 °C were higher than those at 37 °C since AuNPs-D2B were trapped at the cell surface with a higher chance of recognition by the secondary IgG-FITC antibodies, giving a more prominent fluorescence. A comparison between the FL1 intensities obtained at 37 and 4 °C shows almost a constant mean fluorescence intensity (MFI) difference of 20 (*p* < 0.05). This shows that even though saturation was not reached at the maximum concentration used in this study, the rate of internalization was constantly maintained. Until now, only the previously mentioned D2B containing CHNs show a similar flow cytometry approach and comparable finding to ours in terms of PSMA targeting.^25^ However, the other study portraying CS-Ab used flow cytometry to show that the targeting activity of the D2B-coated nanostructures was similar to that of naked D2B.^27^

Although a significant difference was observed between the control and treated cells, a rough MFI of 20 is considered high for a control. A normalized graph for the mean FL1 was plotted to display the results without the background and noise interference (Figure 3c). The modest increment in the signal observed for the control might be due to the variability of the non-specific binding of the secondary antibody FITC-labeled to the cell lines under analysis.^25^

**Figure 5:**
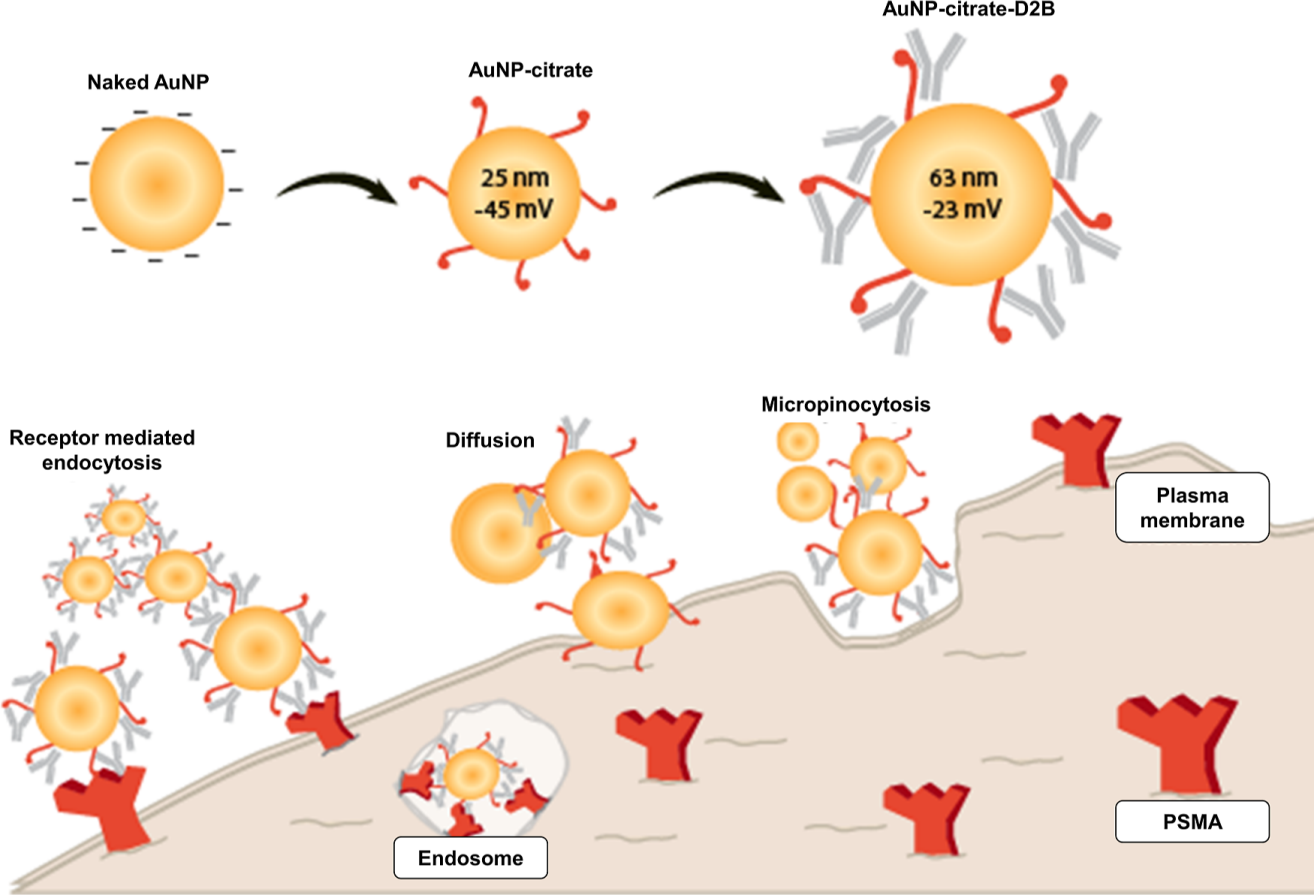
Plausible fate of AuNPs-D2B cellular uptake by PC cells based on its physicochemical properties. Coating AuNPs-citrate with D2B mAb guides its specific binding to its receptor ligand, PSMA, which is highly expressed on PC tumors. This PSMA–antibody complex induces internalization via receptor-mediated endocytosis. Some AuNPs, whether coated or uncoated, can be taken up via the classical macropinocytosis pathway. The efficacy of their cellular uptake is limited by the negative surface charge that is repelled by the negatively charged phospholipid components of the plasma membrane. Abbreviations. AuNPs: gold nanoparticles; mAbs: monoclonal antibodies; PC: prostate cancer; PSMA: prostate specific membrane antigen.

Taken together, the quality control of our synthesized AuNPs-D2B in terms of size, charge, shape, and PSMA targeting efficiency rules out the uptake of the bioconjugates via macropinocytosis or diffusion. Instead, AuNPs-D2B are more likely to be internalized by PC cells via receptor-mediated endocytosis (Figure 5).

Collectively, our data prove that loading D2B onto AuNPs-citrate makes it a desirable carrier for specific drug delivery to PC. Also, our findings confirm that D2B customizes the AuNPs for specific PC-PSMA cell targeting without unwanted cytotoxicity and apoptotic effects. However, it is yet intriguing to compare the targeting efficiency of AuNPs-D2B to PSMA with that of its single-chain variable fragment D2B-AuNPs (AuNPs-scFvD2B), which has not been tested on PC cells. As a substitute, the previous assessment of AuNPs-scFvD2B in terms of human blood stability and immunogenicity suggests that PEGylation is necessary for the in vivo stability of these nanovehicles.^64^ Interestingly, AuNPs-D2B are most likely exempt from PEGylation since it was previously confirmed that mAbs irreversibly adsorb on AuNPs’ surface and are not displaced by blood proteins.^65^

## 5 Conclusions

The milestone of tumor cell-specific multifunctional NPs will enable increased sensitivity to chemotherapeutics with lower systemic toxicity to patients. In this study, we report a simple and unequivocal approach to targeting PC using gold NPs that are conjugated to an anti-PSMA antibody (D2B). This optimized method proved to synthesize non-cytotoxic, non-apoptotic, and target-specific antibody-AuNPs. Therefore, our results promote the potential application of our customized particles to transport therapeutic payloads such as siRNA, chemotherapeutic drugs, and imaging agents.
